# Fungal Resistance to Echinocandins and the MDR Phenomenon in *Candida glabrata*

**DOI:** 10.3390/jof4030105

**Published:** 2018-09-01

**Authors:** Kelley R. Healey, David S. Perlin

**Affiliations:** 1 Department of Biology, William Paterson University, Wayne, NJ 07470, USA; 2 Public Health Research Institute, New Jersey Medical School, Rutgers Biomedical and Health Sciences, Newark, NJ 07103, USA

**Keywords:** *Candida glabrata*, drug resistance, tolerance, FKS, MSH2, echinocandin, azole

## Abstract

*Candida glabrata* has thoroughly adapted to successfully colonize human mucosal membranes and survive in vivo pressures. prior to and during antifungal treatment. Out of all the medically relevant *Candida* species, *C. glabrata* has emerged as a leading cause of azole, echinocandin, and multidrug (MDR: azole + echinocandin) adaptive resistance. Neither mechanism of resistance is intrinsic to *C. glabrata*, since stable genetic resistance depends on mutation of drug target genes, *FKS1* and *FKS2* (echinocandin resistance), and a transcription factor, *PDR1*, which controls expression of major drug transporters, such as *CDR1* (azole resistance). However, another hallmark of *C. glabrata* is the ability to withstand drug pressure both in vitro and in vivo prior to stable “genetic escape”. Additionally, these resistance events can arise within individual patients, which underscores the importance of understanding how this fungus is adapting to its environment and to drug exposure in vivo. Here, we explore the evolution of echinocandin resistance as a multistep model that includes general cell stress, drug adaptation (tolerance), and genetic escape. The extensive genetic diversity reported in *C. glabrata* is highlighted.

## 1. Epidemiology and Mechanisms of Resistance

Invasive fungal infections are a major cause of global morbidity and mortality, accounting for nearly 1.4 million deaths a year [1]. Fungal populations colonize the human host at multiple body sites and represent the majority of eukaryotes in the human gut microbiome, with most organisms having a potential to act as opportunistic pathogens during immunosuppression or when natural barriers are disrupted [2]. Bloodstream fungal infections, largely caused by yeasts of the *Candida* genus, are associated with high mortality rates (45–75%) and pose a serious threat to immunocompromised individuals, including cancer and AIDS patients, organ transplant recipients, and premature infants. The increasing burden of fungal infections has led to a rise in the use of antifungal agents for their treatment and prevention. Unfortunately, treatment options for invasive fungal infections are extremely limited, as there are few antifungal drug classes. For decades, the azole antifungals (e.g., fluconazole), which are fungistatic drugs targeting membrane sterol biosynthesis, were used as primary prophylaxis/therapy to prevent/treat *Candida* infections, with *C. albicans* as the predominant infecting species. But epidemiological shifts in infecting organisms toward non-*albicans Candida* species, which are inherently azole resistant (e.g., *C. krusei*) or rapidly acquire resistance (e.g., *C. glabrata*), has led to the widespread use of echinocandin antifungal drugs.

In most clinical settings, *C. albicans* is the predominant bloodstream pathogen. Yet, the prevalence of *C. glabrata* infections has been rising for several decades and, at 18–25% of *Candida* isolates, it is the second most common *Candida* bloodstream infection in North America. In some settings, such as patients with hematological malignancies, it is the principal bloodstream fungal pathogen [3]. Due to the widespread use of azole antifungals for prophylaxis/therapy, global azole resistance among *C. glabrata* isolates is around 8% [4], while some centers have rates exceeding 20% [5]. Echinocandin therapy is highly efficacious, but emerging echinocandin drug resistance is a growing threat to successful clinical management. Among *C. albicans* and other *Candida* species, the frequency of echinocandin resistance remains relatively low (1–3%) [6,7], but this is not true for *C. glabrata*, where resistance is more severe and often presents as multidrug (MDR) resistance [8,9]. While echinocandin resistance among *C. glabrata* isolates ranges from 3–5% in population-based studies [10], some centers report rates of 10–15% [3,11]. Strains with MDR phenotypes (azole and echinocandin, and sometime polyene resistance) are increasingly encountered with some centers. Nearly one-third of echinocandin resistant isolates are also resistant to azoles [12].

While multiple mechanisms of azole resistance have been reported for *Candida* species [13], the overwhelming singular mechanism of resistance identified in clinical isolates of *C. glabrata* is mutation of the transcription factor *PDR1*, which leads to increased expression of multidrug transporters that act as efflux pumps [14,15]. Unlike azoles, multidrug transporters do not appear to play a role in echinocandin resistance, as echinocandins are not substrates for transport [16]. As such, echinocandins are fully active against azole resistant *Candida* [17].

The echinocandin drugs (caspofungin, micafungin and anidulafungin), which were first approved for clinical use in 2001, target and inhibit the membrane-associated (and fungal specific) β-1-3-d-glucan synthase and block the biosynthesis of β-1,3-glucan, a major structural component of the fungal cell wall. They are broadly active against *Candida* species, in which they are considered fungicidal (more on this later). The enzyme complex consists of a structural/catalytic subunit encoded by *FKS* genes; and its activity is regulated by Rho, a GTP-binding protein [18]. Clinical resistance involves modification of the Fks subunits [19]. In *C. glabrata,* two functionally redundant genes, *FKS1* and *FKS2,* encode glucan synthase catalytic subunits [20]. In most *Candida* species mutations occur in two highly conserved “hot-spot” regions of *FKS1* and, in *C. glabrata*, *FKS2*. Resistance-conferring amino acid substitutions induce elevated MIC values [21] and the most prominent mutations can reduce the sensitivity of glucan synthase (IC_50_) to drug by >3,000 fold [22]. In the 16 years following FDA approval of caspofungin, *FKS* mutations are still the only mechanism associated with clinical failures [10,23]. Given a long clinical history of safe and efficacious therapy, echinocandins are now the IDSA recommended preferred antifungal agent for treatment of candidiasis among high-risk patient populations [24]. 

Echinocandin resistance always arises during therapy and is associated with repeated or chronic drug exposure, although resistance can also follow brief drug exposure [25]. Thus, *C. glabrata* has an elevated potential relative to other *Candida* species to develop echinocandin resistance, for reasons that are currently not understood. The global resistance problem is expected to grow more severe as expanding numbers of patients are exposed to antifungal prophylaxis and echinocandin drugs like caspofungin are now generic. Given the importance of this drug class as a first-line agent, there is an urgent need to better understand factors that contribute to and limit the emergence of echinocandin resistance among patients with *C. glabrata* infections.

## 2. Evolution of Echinocandin Resistance

Clinical antifungal treatment failure is most often a combination of microbial factors, host factors, drug pharmacokinetics (PK)/pharmacodynamics (PD), and drug distribution at the site of infections. All of these factors contribute to therapeutic efficacy and resistance development, although this review will primarily focus on microbial genetic factors contributing to echinocandin resistance. While the terminal step of echinocandin resistance (*FKS* mutation) has been well defined, mechanisms used by *Candida* to survive as both a commensal and an opportunistic pathogen within a harsh environment consisting of bacterial microbiota and host immune factors are less well characterized. All colonizing strains of *Candida* employ mechanisms of adaptation, but *C. glabrata* has a prominent ability to adapt and survive antifungal pressure in vivo, resulting in drug resistance. The emerging pathogen *C. auris* has arisen as a considerable public health concern following reports of elevated rates of antifungal resistance and horizontal transmission within healthcare centers [26]. Conversely, like other *Candida* species, transmission of *C. glabrata* between patients has rarely been reported, suggesting independent development of antifungal resistance within most patients. Unlike *C. albicans*, *C. glabrata* does not normally form hyphae or secrete hydrolytic enzymes, and therefore, elicits a lesser immune response [27]. Despite this apparent lack of virulence factors, *C. glabrata* can robustly replicate and disseminate upon host immunosuppression. The following sections will explore factors that allow *C. glabrata* to adapt to its environment and develop antifungal resistance at higher rates than other species. 

### 2.1. Drug Adaptation Is a Key Intermediate Leading to Echinocandin Resistance

**Figure 1:**
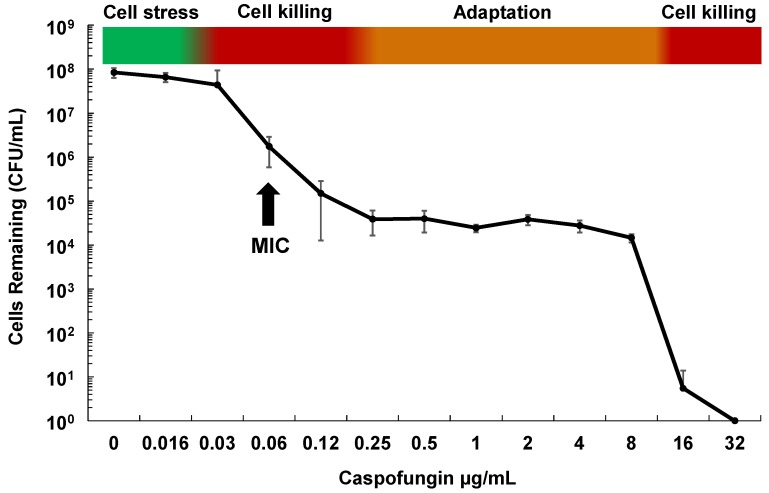
Phases of in vitro cell killing and adaptation with echinocandins and *Candida glabrata*. Cells (1×10^7^) of *C. glabrata* ATCC 2001 were grown in RPMI medium containing caspofungin at the indicated concentrations for 20 h. Dilutions were then plated onto drug free agar-containing plates to determine surviving cell counts. Shown is the average of 4 independent experiments ± standard deviations. The minimum inhibitory concentration (MIC) is indicated for reference.

**Figure 2:**
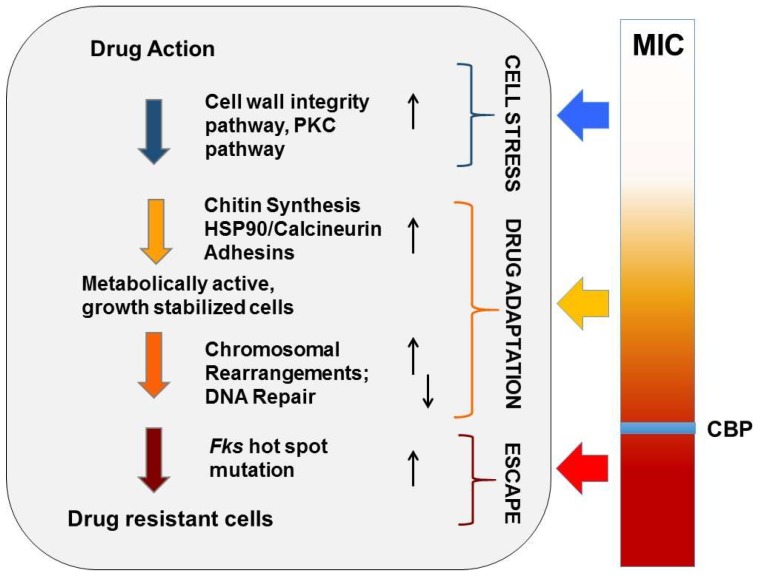
Evolution of echinocandin resistance. Cellular factors that influence the ability of yeast to adapt to echinocandin drug pressure are represented in a multistep model of resistance. Steps include initial cellular stress, drug adaptation, and genetic escape (*FKS* mutation). The clinical breakpoint (CBP) of a species is the MIC measured prior to the formation of *FKS* escape mutants.

Although echinocandins are considered fungicidal drugs in *Candida* species, careful examination of their effect on *C. glabrata* both in vitro and in vivo shows that while the vast majority of cells die upon echinocandin exposure, roughly one in 10^4–5^ of cells survive and demonstrate “drug adaptation” over a wide range of drug exposures (Figure 1). Similarly in an in vivo infection, echinocandin tolerance is manifested as a decline in target organ fungal burdens (e.g., from 10^9^ to 10^4^ cells), but not true sterilization, as fungal stasis is achieved (i.e., no net change cell counts) [28]. Cells that survive echinocandin action (without forming *FKS* mutations) are defined as drug tolerant (or adapted), as they are fully sensitive to drug when re-cultured. They may display higher MIC values but respond to drug in pharmacodynamic models [29]. Ultimately, such adapted cells can persist long enough to give rise to *FKS* mutants, which escape drug action and result in clinical failure (Figure 2). Despite this key role of drug adaptation in development of drug resistance, the factors underlying echinocandin adaptation in *C. glabrata* have not been well defined, particularly in vivo.

One factor that may aid *C. glabrata* in echinocandin adaptation is poor drug penetration into sites of colonization or infection. The echinocandins are intravenously administered drugs that appear to distribute weakly in the GI tract [30]. Some echinocandins, like micafungin, penetrate intraabdominal abscesses of murine models at considerably lower concentrations than what is measured in the blood [31]. Following echinocandin treatment, fungal clearance may be observed in the bloodstream, although cells located at sites of colonization or deep tissue infection have been exposed to lower levels of drug, resulting in a potential reservoir of *FKS* mediated resistance. Subsequent or repeated treatment with an echinocandin can lead to rapid breakthrough [32]. This clinical scenario has been modeled in mice as repeated treatments of caspofungin at 4× the humanized dose increased the frequency of *FKS* mutants formed within the GI tract in a model of colonization [30]. Drug penetration can also be hindered by the formation of a biofilm matrix by *Candida* species [33]. The new echinocandin rezafungin (formerly CD101; Cidara, San Diego, CA, USA) can be administered safely at a considerably higher level and can achieve favorable probabilities of PK-PD target attainment [34], which results in increased efficacy and reduced burden/sterilization at the site of intraabdominal abscesses [31]. Ultimately, a balance between drug concentration and mutant prevention would be best, and targeting drug adaptation mechanisms (see below) in combination with an echinocandin may prove beneficial. These are questions researchers should consider when studying echinocandin adaptation and resistance.

### 2.2. Cellular Drivers of Echinocandin Adaptation

Stress tolerance, including antifungal drug tolerance, has been attributed to the activation of multiple stress response pathways within the yeast cell, including the cell wall integrity pathway/Protein Kinase C (PKC)/mitogen activated protein kinase (MAPK) cascade signaling, Hsp90-dependent calcium/calcineurin signaling, high osmolarity glycerol (HOG) signaling, and the cyclic AMP/Protein Kinase A (PKA) signaling pathway [13]. While these responses have been extensively studied in the model fungus *S. cerevisiae* [35], to which *C. glabrata* is closely related, *C. glabrata*, unlike *S. cerevisiae*, has evolved to survive within the human host. Thus, stress tolerance pathways in *C. glabrata* likely have key differences from those in *S. cerevisiae* to reflect the very different challenges of their environments, and should be validated in animal models of colonization and infection. In general, these stress response pathways seem to be involved in the response to multiple antifungal classes and are sometimes, but not always, conserved across fungi. While stress-triggered changes in transcriptional profiles have been reported in *S. cerevisiae* [36], *C. albicans* [37] and *C. glabrata* [38], the roles of these signaling pathways in *C. glabrata* antifungal drug tolerance have not been systematically investigated. 

As detailed above, echinocandin adaptation in *C. glabrata* is a key step towards development of *FKS* escape mutations (Figure 2). Echinocandins target the fungal cell wall. It has been well established that in response to cell wall damage, fungi upregulate a number of stress responses and cell wall maintenance pathways that help the cells tolerate and survive the stress [39]. Of particular importance upon echinocandin exposure is the cell wall integrity pathway which regulates glucan synthesis through Rho1 and cell wall repair. Rho1 activation leads to upregulation of the *FKS* genes and activation of PKC. Cells with decreased PKC activity or those lacking activated MAP kinases (e.g., Sc*BCK1*, Sc*SLT2,* Ca*MKC1*) are hypersensitive to the echinocandins [40,41,42].

### 2.3. *C. glabrata* Specific Echinocandin Adaptation

Some of the stress induced mechanisms mentioned above, such as the cell wall integrity pathway (e.g., *WSC1*, *MKK1*, *BCK1*, *SLT2*) [43,44,45], Hsp90 and calcineurin signaling [46,47], and chromatin remodeling [48,49], have been shown to abrogate echinocandin tolerance or adaptation when disrupted or targeted in *C. glabrata*. In *S. cerevisiae* and *C. albicans*, echinocandin-induced *PKC1* expression has been linked to increased production of cell wall components chitin and mannan, potentially compensating for the loss of β-glucans [40,50,51]. In *C. glabrata*, the significance of chitin during echinocandin exposure seems to be more complicated. While one study reported that an increase in chitin led to incomplete killing of *C. glabrata* by caspofungin [45], another reported that there were no significant increases in chitin production upon caspofungin exposure in vitro [52]. A more recent study noted an increase in *C. glabrata* chitin levels upon murine GI tract colonization [53]. We have shown that treatment of colonized mice with a combination of caspofungin and the chitin synthase inhibitor Nikkomycin Z caused an increase in killing of *C. glabrata* within the murine GI tract and a decrease of dissemination upon immunosuppression [30]. In addition to the apparent compensation for β-glucan loss, recent studies have also noted the elevated expression of specific genes (e.g., *BGL2*, *XOG1*, *GAS2*) that are related to the replacement of β-1,3-glucans in the biofilm matrix following echinocandin exposure [54,55], which may also influence adaptation.

In a comprehensive study by Schwarzmuller and colleagues [44], a partial *C. glabrata* gene knockout library was constructed and screened for increased susceptibilities to antifungals, including caspofungin. Multiple gene knockouts, including those involved in cell wall organization, chromatin assembly, transcriptional regulation, and signal transduction, were associated with caspofungin hypersensitivity [44]. Many of these genes have not been linked to echinocandin hypersensitivity in *S. cerevisiae* or *C. albicans*, although for most, it remains to be shown if targeting these cellular pathways/components would negate echinocandin adaptation in vivo. Another important study analyzed genome mutations throughout the echinocandin treatment course of a patient with recurrent *C. glabrata* candidemia [46]. Tracking the progression of *Candida* prior to the acquisition of an *FKS* mutation will begin to shed light on factors essential for echinocandin adaptation. 

### 2.4. Echinocandin- and *FKS* Gene- Specific Effects

Different echinocandins may elicit varying or different fungal adaptive responses. For example, targeting specific sphingolipid biosynthesis genes or chemically altering the sphingolipid cellular makeup led to a differential echinocandin susceptibility pattern in *Candida* species, including *C. glabrata* [56,57]. Although, this differential activity may be due to the physical interaction between the echinocandins and the target Fks proteins within the membrane, potential echinocandin-specific effects should be considered when attempting to “target” an adaptive response mechanism. New glucan synthase targeting echinocandins that are in development may also produce differing cellular responses. As stated above, rezufungin can reportedly penetrate into deep tissue lesions better than micafungin [31] and exhibits a long half-life in PK studies [58,59]. An orally-active glucan synthase inhibitor, SCY-078 (Scynexis, Jersey City, NJ, USA), exhibits activity against some otherwise-resistant *FKS* mutants [60], likely a result of a slightly different binding spot on the Fks protein [61].

As detailed above, genetic resistance to echinocandins requires the formation of mutations within “hotspot” regions of glucan synthase subunits, encoded by *FKS* genes. Most *Candida* species rely on one essential *FKS* gene (*FKS1*), while *FKS2* and *FKS3* are expressed at lower levels and have yet to be fully characterized in *C. albicans*. In *S. cerevisiae*, *FKS2* and *FKS3* are important during sporulation and mating [62,63]. Interestingly, a recent study demonstrated that expression of *FKS2* and *FKS3* in *C. albicans* can influence overall drug sensitivity [64]. *C. glabrata* is the only *Candida* species that has two seemingly redundant, yet differentially regulated, *FKS* subunits: *FKS1* and *FKS2* (this is also true in *S. cerevisiae*). Unlike *S. cerevisiae*, sporulation and mating have not been observed in *C. glabrata* yeast. *FKS2* expression is dependent upon the calcium/calcineurin/Hsp90 signaling pathway, and targeting of this pathway either genetically or chemically results in a reversal of Fks2-mediated resistance in *C. glabrata* [20,46]. While *FKS2* expression was increased following caspofungin or calcium exposure, the authors concluded that transcriptional control was not the only mechanism of Fks2 modulation in *C. glabrata* [20]. Gaining a better understanding of how each *FKS* gene is controlled, transcriptionally and otherwise, will help tease out one more unique property of *C. glabrata* and the response to echinocandins.

### 2.5. MDR, *PDR1* and Adhesins

*Candida glabrata* readily forms MDR phenotypes, which involves separate resistance mechanisms for each drug class (modification of drug target site for echinocandins versus expression of drug efflux transporters for azoles). Despite the apparent lack of mechanistic overlap, a nexus may exist. The presence of a *PDR1* mutation appears to increase the ability of *C. glabrata* to adapt to other stressors, including echinocandin exposure. Specific *PDR1* mutations in *C. glabrata* not only confer azole resistance, but can also enhance adhesion to epithelial cells through increased expression of the epithelial adhesin gene *EPA1* [65,66,67,68]. The genome of *C. glabrata* carries a large number of *EPA* (epithelial adhesin) genes that encode for adhesin proteins [69,70,71]. Interestingly, a recent study found that separate clinical isolates expressed a unique variety of adhesins and other cell wall proteins [72], most likely due to the subtelomeric positions of adhesin genes and the unusually high genomic plasticity of *C. glabrata* [70,73] (see more below). *PDR1*-mediated increased expression of *EPA1* increased organ colonization in a mouse UTI model [66] and virulence in a model of hematogenous disseminated candidiasis [74,75]. An increase in adhesion that aids in colonization of mucosal membranes may also increase echinocandin tolerance through common cellular pathways (Figure 2). Again, the expansion and dissemination of *C. glabrata* is dependent upon the host’s immune response, and this is highlighted by the ability of natural killer (NK) cells to recognize *C. glabrata* through binding of Epa proteins [76].

### 2.6. Exploiting Genetic Diversity

According to classical evolution, random mutations arise in microbial populations, whereupon a change in conditions (e.g., exposure to antifungal drug) favors pre-existing mutants that are more fit under the new conditions (i.e., resistant to the drug). However, an extensive body of work in bacteria [77,78,79,80] and *S. cerevisiae* [81], as well as computational models of mutation rates [82], indicates that in stressed cells genome maintenance and repair mechanisms are altered, promoting mutability and increasing the pool of genetic diversity from which drug-resistant mutations can emerge. Furthermore, heteroresistance may play a vital role in cellular adaptation during stress, and epigenetic and post-translational modification mechanisms are emerging [83,84]. Such mechanisms may be particularly important for haploid organisms like *C. glabrata* that have extremely limited ability to generate genetic diversity via meiosis and recombination [85]. Thus, the probability that a tolerant *C. glabrata* cell will genetically escape drug action is a function of its mutagenic potential (Figure 2). However, the mechanisms of mutagenesis operating in drug-tolerant *C. glabrata* cells are not fully known.

The ability to increase genetic diversity within a *C. glabrata* population would help the yeast survive as a commensal and transition into a pathogen. Several studies, including ours, have shown that clinical isolates of *C. glabrata* show astounding genetic diversity both in terms of nucleotide sequence and chromosome structure [86,87,88,89]. *C. glabrata* can seemingly duplicate and reorganize chromosomes at high frequencies generating changes in size and variation of chromosomes [88,90]. As a result, studies have identified gene duplications in *C. glabrata* to include that of cell wall proteins, such as mannosyltransferases, aspartyl proteases, phospholipases, the ABC transporter *PDH1*, and the sterol transporter *AUS1* [88,90]. Additionally, as mentioned earlier, *EPA* adhesin genes important for mucosal colonization have also been heavily duplicated within *C. glabrata* genomes [72,90]. It is not clear whether these rearrangements occur acutely in response to treatment and/or represent divergent sub-species best adapted for colonization. Variations in karyotypes were identified in clinical isolates taken from the same patients over the course of antifungal treatment [86,91,92]; however, we have also found that that different sequence types (STs), or clades, are characterized not only by different single nucleotide polymorphisms (SNPs) but also by varying chromosomal configurations [86]. While there is a high correlation between chromosomal configurations and STs, it is not absolute. 

Are chromosomal integrity components in *C. glabrata* missing or downregulated? According to Polakova and colleagues [88], homologs of two proteins (Ten1 and Rif2) that function in *S. cerevisiae* telomere length end protection and length regulation are absent from the *C. glabrata* genome, although additional homologs with similar functions, such as Rap1, Sir3, and Rif1, have been characterized in *C. glabrata* [73,93]. Expression of the adhesin genes is regulated by several subtelomeric silencing complexes (see [72] for review). The extensive chromosomal rearrangements between strains have been a partial barrier to rapid Illumina whole genome sequencing of *C. glabrata* clinical isolates because the reference strain ATCC 2001, which belongs to ST15, cannot serve as an appropriate template for assembly of genomes of many other STs. Overcoming these technical difficulties will aid in the understanding of *C. glabrata* drug adaptation through chromosomal rearrangement. 

Fungi contain multiple mechanisms that regulate mutagenesis, including several highly-conserved DNA repair systems, such as double-strand break repair (DSBR), base-excision repair (BER), nucleotide-excision repair (NER), post-replication repair (PRR), and mismatch repair (MMR). DNA polymerases, including several error-prone polymerases, also impinge on mutation rates [94,95,96]. Defects or programmed changes (e.g., as during stress-induced mutagenesis [77]) in these mechanisms are often associated with increased mutation rates [97]. These pathways have been well studied in vitro in the model fungus *S. cerevisiae*. We have previously evaluated the role of MMR in *C. glabrata* and shown that active MMR suppresses emergence of drug-resistant mutants and that naturally occurring variants in *C. glabrata* MMR gene *MSH2* may promote development of resistance in some clades [87,98].

Importantly, we and others have found that different *MSH2* genotypes are characteristic of distinct STs/clades, suggesting that different STs may have different propensity towards mutability and acquiring drug resistant gene variants [86,98,99,100]. This is significant because the distribution of *C. glabrata* STs varies both by geography and over time. For instance, *C. glabrata* ST distribution in Atlanta area hospitals changed significantly between 1992 and 2008, the time period that includes the introduction of echinocandins [101]. One significant change is the increased prevalence of ST16, which carries a *msh2* variant associated with increased echinocandin resistance frequencies in vitro [87] and was shown to be more prevalent among drug-resistant clinical isolates [102], suggesting that this ST may have an increased capacity for drug escape. However, there are also expanding STs (e.g., ST3) that do not carry specific *msh2* alterations, emphasizing that there are additional factors at play. 

Specific *MSH2* alleles most likely diversify populations of *C. glabrata* to better survive in vivo, and upon prolonged antifungal exposure, may aid in drug target mutation. Multiple clinical studies performed at non-U.S. clinics have reported no correlation between *MSH2* genotype and clinical resistance frequencies in populations with limited drug exposure and/or very low levels of drug resistance [98,99,100,103]. DNA repair alterations may be more relevant in certain populations where antifungals are routinely used for prophylaxis and treatment, and where a higher prevalence of MDR phenotypes are observed [87]. It should be noted that not all *MSH2* mutations lead to significant increases in mutants in vitro; for example, alleles encoding for P208S/N890I and E231G/L269F produced greater frequencies of resistant mutants in vitro, while others produced smaller or no increases in frequencies [98,103].

**Figure 3:**
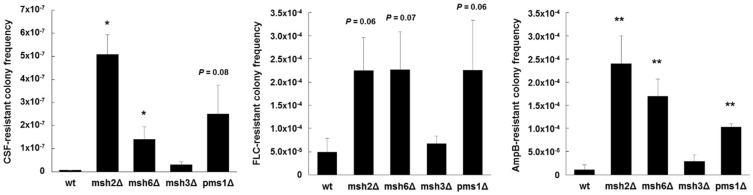
Echinocandin, azole, and polyene resistant colony frequencies of *C. glabrata* mismatch repair deletion strains. Strains were selected agar plates containing 1 µg/mL of caspofungin (CSF), 256 µg/mL of fluconazole (FLC), or 2 µg/mL of amphotericin B (AmpB) (panels left to right) (all concentrations 8-16x the corresponding MIC). Dilutions were plated onto drug-free media to determine exact CFU counts. Frequencies were calculated as the number of colonies on the drug plate divided by the total CFU plated. Frequency averages were calculated from at least three independent selections. * *p* < 0.05 and ** *p* < 0.01 (student’s *t*-test; two-tailed).

Additional mechanisms at play within individual isolates exhibiting the same *MSH2* genotype also likely affect the mutagenic properties. For example, when we expressed a wild type copy of *MSH2* in several strains that contained deficient *MSH2* alleles, an increase in *FKS* mutagenesis was complemented in some strains, but not in others [104], indicative of additional mechanisms of mutagenesis at play. Importantly, *MSH2* likely represents one piece in a multifaceted and complex puzzle that makes up drug escape. Our preliminary studies also show that disruption of other genes involved in MMR, such as *PMS1* and *MSH6*, produce greater frequencies of antifungal-resistance and *FKS* mutagenesis in vitro (Figure 3). How sequence polymorphisms or transcriptional control of these genes affects *C. glabrata* is unknown. As listed above, additional cellular mechanisms may also influence mutagenesis in *C. glabrata* and ultimately affect its ability to colonize, disseminate, and develop resistance. In a broader context, defects or changes in DNA repair may be an evolutionarily adaptive mechanism(s) of *C. glabrata* to generate greater genetic diversity among colonizing strains in order to better adapt to its environment, and introduction of antifungal drug into that environment is just one more factor in a slew of others. Notably, the consequences of this genetic diversity on colonization, infection, and drug resistance are not fully understood. A more dynamic view of cellular mutagenic potential may be more relevant that individual components. This will require new tools and approaches.

## 3. Conclusions

Rates of acquired resistance to azoles and echinocandins are substantially higher among strains of *Candida glabrata* compared to other *Candida* species. The ability of *C. glabrata* to survive antifungal pressure at high rates within individual patients highlights its astounding adaptive flexibility. This flexibility is likely due to a myriad of factors, including strong general cell stress responses (e.g., cell wall integrity pathway and regulation of associated genes) and multiple mechanisms of drug adaptation (e.g., HSP90/calcineurin, chitin synthesis, adhesion, genetic diversity). The combination of these cellular mechanisms (and other factors such as host immune status and drug penetration and pharmacokinetics) ultimately permit or enhance genetic escape (*PDR*1 and *FKS* mutations) and stable resistance, which can result in clinical failure. Importantly, how many of these factors influence colonization, infection, and drug resistance in vivo have not been fully determined.
